# Supplementary material for: Tailoring Coherent Microwave Emission from a Solid‐State Hybrid System for Room‐Temperature Microwave Quantum Electronics
Source: Adv Sci (Weinh). 2024 Jul 15;11(35):2401904. doi: 10.1002/advs.202401904 (PMC11425272; doi:10.1002/advs.202401904)
Supplement: Supplementary file 1 — Supporting Information [file ADVS-11-2401904-s001.docx]

Supporting Information:

TAILORING COHERENT MICROWAVE EMISSION FROM A SOLID-STATE HYBRID SYSTEM FOR ROOM-TEMPERATURE MICROWAVE QUANTUM ELECTRONICS

Kaipu Wang^†^, Hao Wu^†*^, Bo Zhang, Xuri Yao, Jiakai Zhang, Mark Oxborrow, and

Qing Zhao^*^

*Corresponding author. Email: hao.wu@bit.edu.cn, qzhaoyuping@bit.edu.cn.

^†^These authors contributed equally to this work

**Supporting Section 1: Experimental measurement circuits**


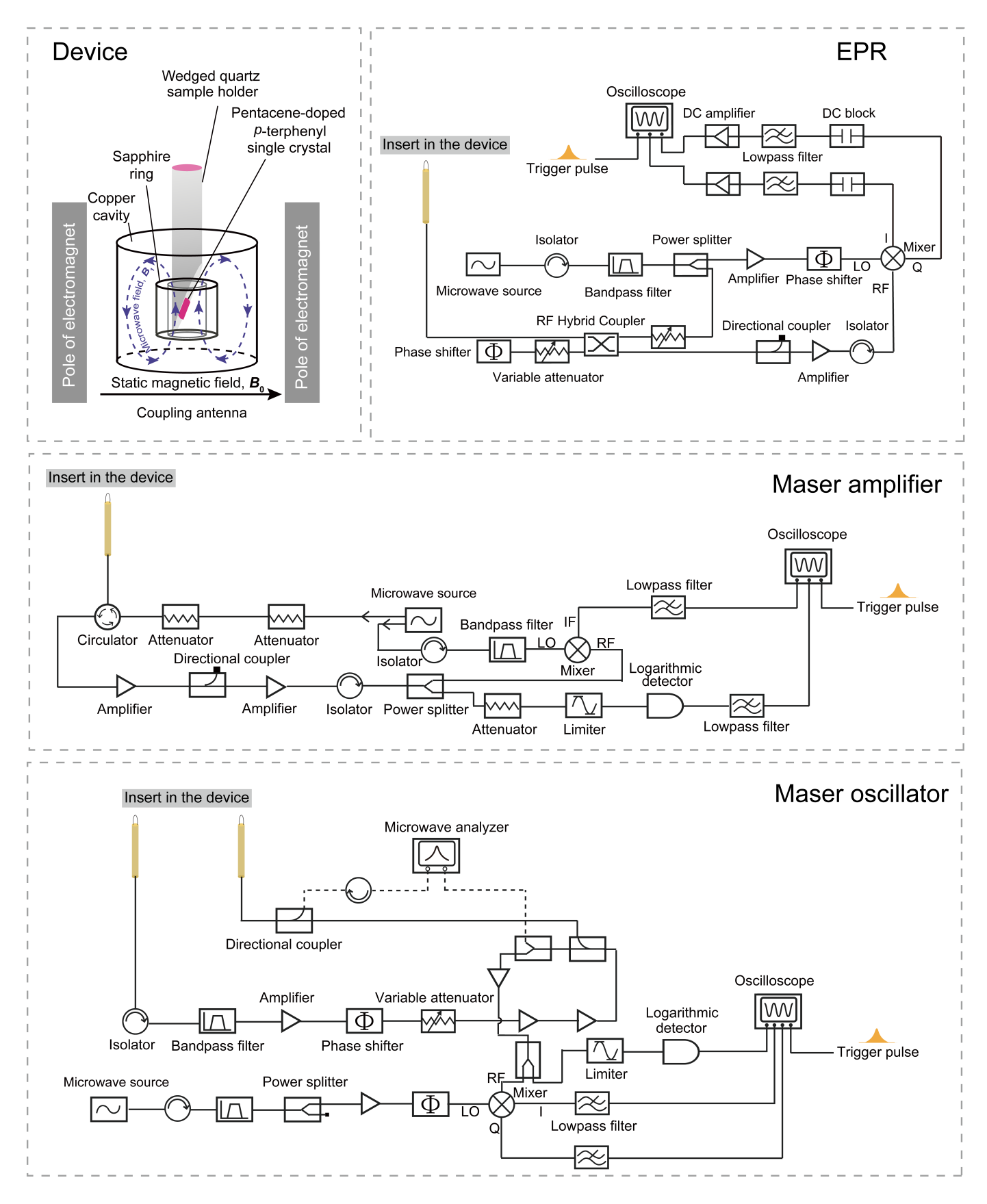


**Supporting Figure 1.** **Experimental setups.**

**Supporting Section 2: Determination of angles $\alpha$ and** $\beta$

As mentioned in the main text, the values of $\alpha$ and $\beta$ can be calculated according to

$\alpha=90^{^{\circ}}-\angle c'\text{Z}_{1}$ (1)

$\beta=\arctan\text{ }\left(\frac{\cos(\angle aZ_{1})}{\cos(\angle bZ_{1})}\right)$ (2)

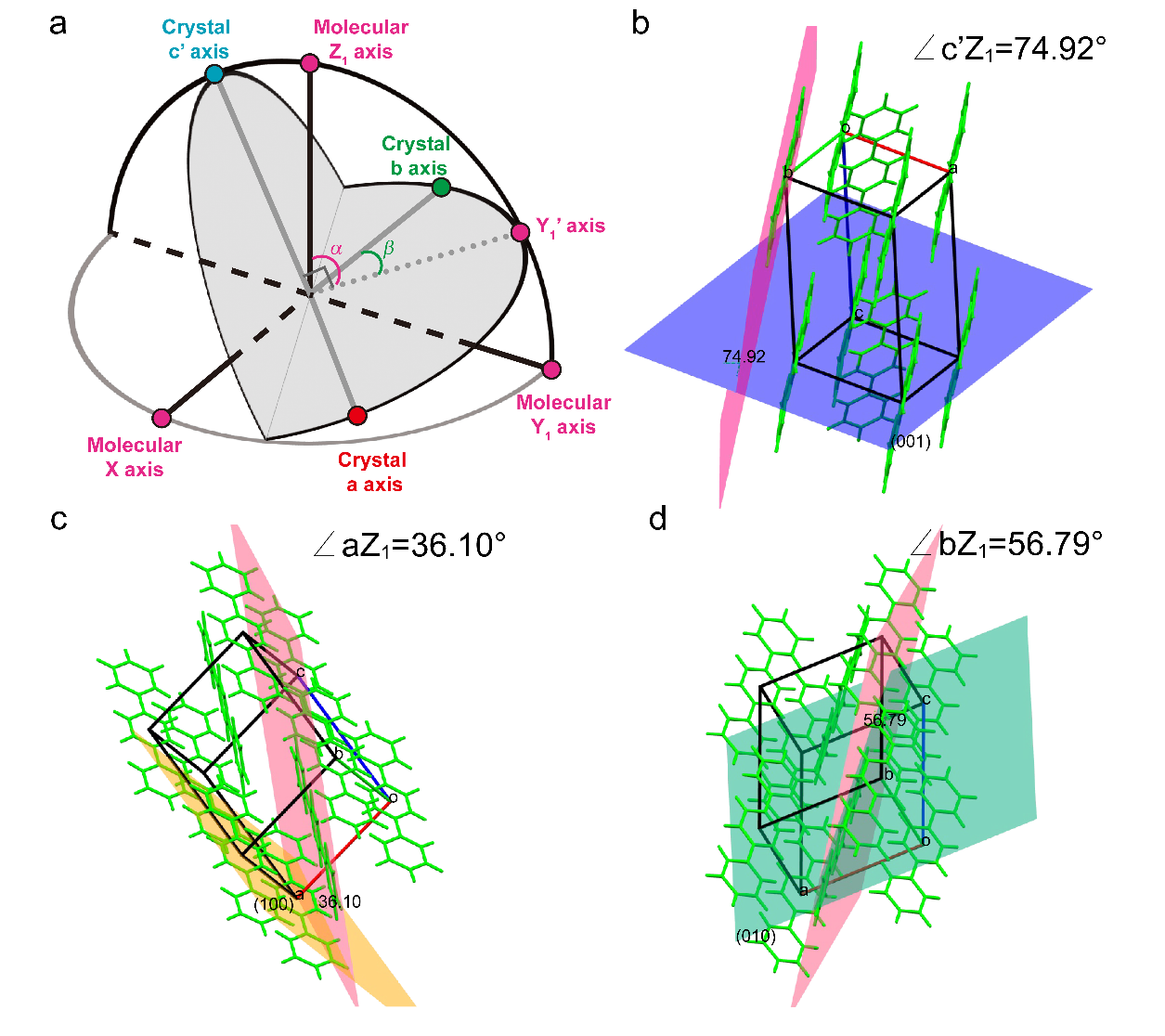
Therefore, we need to obtain the values of $\angle c'Z_{1}$,$\angle aZ_{1}$and$\angle bZ_{1}$. To help visualize the above angles, we reuse the Fig.2c in the main text here as Supporting Fig. 2a. To this end, we employ the Mercury software and the reported crystallography data of *p*-terphenyl^[1]^ to measure the angles between the associated planes to which the above axes are perpendicular. The results are shown in Supporting Fig. 2b-d. By inserting the obtained angles in the above equations, the values of $\alpha$ and $\beta$ can thus be determined to be $15.1^{\circ}$ and $124^{\circ}$, respectively.

**Supporting Figure 2. Angle measurements. a** Angular relationship between the crystallographic and molecular axes. **b**-**d** Measurements of the angles $\angle c'Z_{1}$, $\angle aZ_{1}$ and $\angle bZ_{1}$ using the Mercury software.

**Supporting Section 3: Simulation of the rotation pattern**

The rotation pattern of the EPR spectra shown in the main text was simulated using the pepper function in EasySpin.^[2]^ EasySpin defines a set of standard Cartesian frames. During the simulation, the laboratory, molecular and crystal frames were employed to understand the orientation of the crystal lattice in the spectrometer and the orientation of the pentacene molecules in the *p*-terphenyl crystal. According to the default settings of EasySpin, the laboratory frame’s three unit vectors were expressed as (X_L_, Y_L_, Z_L_), in which Z_L_ is horizontal, along the static magnetic field, and X_L_ is aligned with the microwave field in the resonator, thus vertical. Since the XY_1_ molecular plane of pentacene-doped *p*-terphenyl is parallel to the static magnetic field, and X, Y_1_, and Z_1_ in the molecular frame are perpendicular to each other, we could obtain that the Euler angle from the molecular frame to the laboratory frame is [-90 270 0]. Subsequently, the rotation matrix of the corresponding transformation could be obtained through the Euler angle. Supporting Table 1. shows the angles and cosines between the molecular axes (X, Y_1_, Z_1_) and the crystallographic axes (a, b, c’) of *p*-terphenyl, measured using the Mercury software.

**Supporting Table 1. Angles and cosines**

|  | $\angle aX$ | $\angle bX$ | $\angle c'X$ | $\angle aY_{1}$ | $\angle bY_{1}$ | $\angle c'Y_{1}$ | $\angle aZ_{1}$ | $\angle bZ_{1}$ | $\angle c'Z_{1}$ |
| --- | --- | --- | --- | --- | --- | --- | --- | --- | --- |
| Angle | 73.08 | 89.62 | 164.75 | 59.04 | 32.67 | 81.26 | 36.10 | 123.21 | 74.92 |
| Cosine of the angle | 0.29 | 0.01 | -0.96 | 0.51 | 0.84 | 0.15 | 0.81 | -0.55 | 0.26 |

The rotation matrix $R$ could thus be obtained through the cosine values. Note that, there might be an ambiguity of an angle of $\pi$ for the actual sample mounting, which could be confirmed by the trEPR measurements.

$$R=\left(\begin{matrix} \cos(\angle\text{aX}) & \cos(\angle\text{bX}) & \cos(\angle c\text{'}X) \\ \cos(\angle\text{aY}_{1}) & \cos(\angle\text{bY}_{1}) & \cos(\angle c\text{'Y}_{1}) \\ \cos(\angle\text{aZ}_{1}) & \cos(\angle\text{bZ}_{1}) & \cos(\angle c'\text{Z}_{1}) \end{matrix}\right)$$

According to eulang($R$) $\times$ 180/$\pi$ in Matlab, we obtain the Euler angle of the rotation from the crystal frame (a, b, c’) to the molecular frame (X, Y_1_, Z_1_) which is [326.12 75.70 9.28] $\times$ $\pi$/ 180. Multiplication of the two derived rotation matrices could provide the rotation matrix for the transformation from the crystal frame to the laboratory frame, and then the Euler angle of the transformation could be obtained. On the other hand, for the simulation parameters associated with the pentacene triplet spins, the zero-field splitting parameters $D=1395.57$MHz and $E=-53.35$MHz^[3]^ were employed. The spectrum rotating around the laboratory X_L_ could be simulated by Easyspin. Due to the uncertainties of the angles measured using the Mercury software and the sample mounting, we found the fine adjustment of the Euler angle of the rotation from the crystal frame (a, b, c’) to the molecular frame (X, Y_1_, Z_1_) to [326.27 76.445 10.07] $\times$ $\pi$/180 could offer the better match of the simulated rotation pattern to the experimental results.

**Supporting
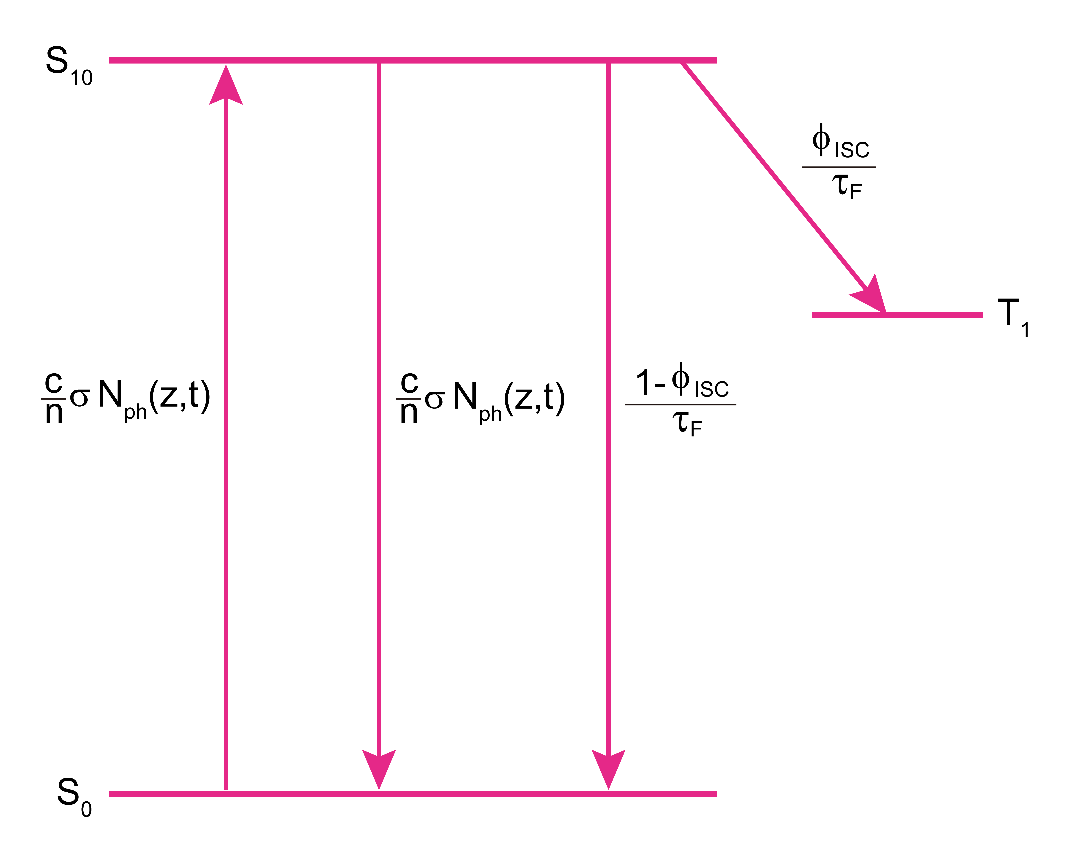
Section 4: The light propagation theory**

**Supporting Figure 3. Simplified Jablonski diagram and the associated dynamics upon photoexcitation of pentacene molecules.** The system is simplified to be a three-level system which involves the singlet ground state S_0_, the singlet excited state S_10_ and the triplet state T_1_. The dynamics processes among the electronic states are the optical pumping, the stimulated emission, the spontaneous emission and the intersystem crossing. The rates of the former two processes can be assumed to be the same^[4]^ and associated with the time - and penetration-depth-dependent optical photon densities as well as the refractive index and the absorption cross section of the crystal. The rates of the later two processes depend on the intersystem crossing yield and the fluorescence lifetime. The decay from T_1_ to S_0_ is neglected in the model because it is much slower compared to other processes especially for the nanosecond optical pumping.

In order to simulate the quantum amplification process, the simplified light propagation theory,^[5-6]^ based on the model shown in Supporting Fig.3 and the derived coupled differential equations (Supporting Eqs. 3-7), was employed to quantitatively analyze the relationship between the triplet spin densities and the penetration depth $z$ under a certain pump intensity at the short time scale $t$ of applied light pulses.

$\frac{\partial N_{\text{T}}(z,t)}{\partial t}=\frac{\Phi_{\text{ISC}}}{\tau_{\text{F}}}N_{10}(z,t)\text{ }$ (3)

$\begin{matrix} \frac{\partial N_{0}(z,t)}{\partial t}=-\frac{c}{n_{\alpha}}\sigma_{\alpha}N_{\text{ph}\alpha}(z,t)N_{0}(z,t)-\frac{c}{n_{\gamma}}\sigma_{\gamma}N_{\text{ph}\gamma}(z,t)N_{0}(z,t)+ \\ \left(\frac{c}{n_{\alpha}}\sigma_{\alpha}N_{\text{ph}\alpha}(z,t)+\frac{c}{n_{\gamma}}\sigma_{\gamma}N_{\text{ph}\gamma}(z,t)+\frac{1-\Phi_{\text{ISC}}}{\tau_{\text{F}}}\right)N_{10}(z,t) \end{matrix}$ (4)

$\begin{matrix} \frac{\partial N_{10}(z,t)}{\partial t}=\frac{c}{n_{\alpha}}\sigma_{\alpha}N_{\text{ph}\alpha}(z,t)N_{0}(z,t)+\frac{c}{n_{\gamma}}\sigma_{\gamma}N_{\text{ph}\gamma}(z,t)N_{0}(z,t)- \\ \left(\frac{c}{n_{\alpha}}\sigma_{\alpha}N_{\text{ph}\alpha}(z,t)+\frac{c}{n_{\gamma}}\sigma_{\gamma}N_{\text{ph}\gamma}(z,t)+\frac{1}{\tau_{\text{F}}}\right)N_{10}(z,t) \end{matrix}$ (5)

$\frac{\partial N_{\text{ph}\alpha}(z,t)}{\partial t}+\frac{c}{n_{\alpha}}\frac{\partial N_{\text{ph}\alpha}(z,t)}{\partial z}=-\frac{c}{n_{\alpha}}\sigma_{\alpha}N_{\text{ph}\alpha}(z,t)(N_{0}(z,t)-N_{10}(z,t))$ (6)

$\frac{\partial N_{\text{ph}\gamma}(z,t)}{\partial t}+\frac{c}{n_{\gamma}}\frac{\partial N_{\text{ph}\gamma}(z,t)}{\partial z}=-\frac{c}{n_{\gamma}}\sigma_{\gamma}N_{\text{ph}\gamma}(z,t)(N_{0}(z,t)-N_{10}(z,t))$ (7)

$N_{\text{T}}$, $N_{0}$and $N_{10}$ are the spin densities in the triplet state (i.e. T_1_), the singlet ground state and the singlet excited state. Since the crystal is birefringent, the laser beam is split into two rays - a fast ray and a slow ray, with the associated photon densities $N_{\text{ph}\alpha}$ and $N_{\text{ph}\gamma}$. The corresponded refractive indices are $n_{\alpha}$ and $n_{\gamma}$,^[7]^ and the absorption cross sections are $\sigma_{\alpha}$ and $\sigma_{\gamma}$, respectively. $c$ is the speed of light in vacuum. $\Phi_{\text{ISC}}$ is the ISC yield^[8]^ and $\tau_{\text{F}}$ is the fluorescence lifetime^[9]^ for the pentacene molecules doped in *p*-terphenyl. The values and expressions of the above quantities are summarized in Supporting Table 2.

**Supporting Table 2. Parameter values and expressions employed in the simulation**

| Parameter | $n_{\alpha}$ | $n_{\gamma}$ | | $\Phi_{\text{ISC}}$ | $\tau_{\text{F}}$ | | $\frac{\alpha_{\text{p x}}\omega}{4\pi\varepsilon_{0}c}$ | $\frac{\alpha_{\text{p y}}\omega}{4\pi\varepsilon_{0}c}$ |
| --- | --- | --- | --- | --- | --- | --- | --- | --- |
| Value | 1.584 | 2.004 | | 62.5 | 9 ns | | $0.53\times10^{-18} \text{cm}^{2}$ | $0.29\times10^{-18} \text{cm}^{2}$ |
| Parameter | | | $\sigma_{\alpha}$ | | | $\sigma_{\gamma}$ | | |
| Expression | | |  | | | $\frac{1}{9\varepsilon_{0}}\left[\frac{(n_{\gamma}^{2}+2)^{2}}{n_{\gamma}}\right]\alpha_{\text{p x}}\frac{\omega}{c}$ | | |

Where $\varepsilon_{0}$ is the permittivity of free space, $\omega$ is the angular frequency of the pump light, is the angle between the b-axis and the Y_1_, which is approximately $33^{\circ}$. $\alpha_{\text{p x}}$ and $\alpha_{\text{p y}}$ are the diagonal elements of the molecular polarizability tensor of pentacene^[6]^. By inserting the values of the parameters in the above coupled differential equations for the numerical calculations via Mathematica, the total number of the triplet spins (the sum of the spin populations in T_-1_, T_0_ and T_+1_) generated by the optical pumped can be obtained by integrating N_T_(z, t) over the penetration depth and then multiplying with the laser pumped area of the crystal (1 mm$\times$ 2 mm). Apparently, the laser pumped area can be improved in the future for gaining more triplet spins.

**References**

[1] A. P. Rice, F. S. Tham, E. L. Chronister, *J. Chem. Crystallogr.* **2013**, 43, 14.

[2] S. Stoll, A. Schweiger, *J. Magn. Reson.* **2006**, 178, 42.

[3] T. C. Yang, D. J. Sloop, S. I. Weissman, T. S. Lin, *J. Chem. Phys.* **2000**, 113, 11194.

[4] H. J. Carmichael, *Statistical methods in quantum optics 1: master equations and Fokker-Planck equations*, Springer Science & Business Media, **2013**.

[5] Y. Quan, *Development of Triplet Dynamic Nuclear Polarization for Polarization Analysis in Small-Angle Neutron Scattering.* Ph.D. thesis, Universität Basel, **2021**.

[6] Y. F. Quan, N. Niketic, J. M. Steiner, T. R. Eichhorn, W. T. Wenckebach, P. Hautle, *Mol. Phys.* **2023**, 121, 13.

[7] K. S. Sundararajan, *Z. Kristall.* **1936**, 93, 238.

[8] K. Takeda, K. Takegoshi, T. Terao, *J. Chem. Phys.* **2002**, 117, 4940.

[9] F. G. Patterson, H. W. H. Lee, W. L. Wilson, M. D. Fayer, *Chem. Phys.* **1984**, 84, 51.
